# Supplementary material for: MetaRibo-Seq measures translation in microbiomes
Source: Nat Commun. 2020 Jun 29;11:3268. doi: 10.1038/s41467-020-17081-z (PMC7324362; doi:10.1038/s41467-020-17081-z)
Supplement: Supplementary file 10 — Supplementary Data 7 [file 41467_2020_17081_MOESM10_ESM.zip › File2/Confidence_High_Taxonomy/378324_out.krona.html]

Javascript must be enabled to view this page.

members
magnitude
magnitudeUnassigned
count
unassigned
taxon
rank

378324\_out

5

2759
superkingdom
1

4751
kingdom
1

1
subkingdom
451864

1
4890
phylum

147537
subphylum
1

4891
class
1

1
4892
order

1
1156497
family

4919
genus
1


SRS023914\_contig\_number\_contig-100\_1300.161646
species
53655
1

4

SRS013687\_contig\_number\_contig-100\_1571.179844SRS051031\_contig\_number\_contig-100\_1391.196250SRS098571\_contig\_number\_contig-100\_1422.365353SRS1041145\_contig\_number\_contig-100\_1416.197434
